# TP-CanineNet: Temporal Context Contrastive Learning with Pseudo-Label Supervision for Abnormal Behavior Detection of Canine

**DOI:** 10.3390/ani16131997

**Published:** 2026-06-29

**Authors:** Xiangyun Guo, Xiaoya Kong, Chuiyu Kong, Jiashuo Feng, Yuxin Liu

**Affiliations:** 1 College of Management Science and Engineering, Beijing Information Science & Technology University, Beijing 102206, China; guoxy@bistu.edu.cn (X.G.); fjshhxx@163.com (J.F.); liuyuxin6409@163.com (Y.L.); 2 College of Engineering, China Agricultural University, Beijing 100193, China; chuiyukong99@163.com

**Keywords:** canine, temporal context modeling, contrastive learning, pseudo-label, behavior recognition

## Abstract

Canines exhibit various behavioral abnormalities, such as excessive barking, destructive behaviors, and indoor defecation when left at home alone. Identifying these abnormal behaviors and implementing scientific and reasonable interventions can help improve canine welfare and promote harmonious coexistence between humans and companion animals. However, existing canine behavior recognition methods struggle to adapt to the characteristics of strong temporal continuity and uneven motion amplitude of abnormal behaviors exhibited by lonely dogs, resulting in inadequate temporal feature representation and low recognition accuracy. Therefore, this study developed a TP-CanineNet model based on a Weakly Supervised Video Anomaly Detection (WS-VAD) framework to address this issue. The model integrated a Temporal Context Aggregation (TCA) module to efficiently capture local–global temporal dependencies and suppress temporal noise, and further enhances the representation of temporal features in dog behaviors. Meanwhile, a Pseudo-Instance Discriminative Enhancement (PIDE) module is adopted to strengthen the feature distinction between abnormal and normal behaviors. We constructed an Alone-Dog dataset comprising 430 video samples and 60 ground-truth labeled samples to validate the model’s effectiveness. Experimental results showed that the proposed model achieved a frame-level AUC of 85.19% and an AP of 72.55%, representing improvements of 2.20% and 8.33%, respectively, over the baseline model. The method can provide intelligent detection of domestic dog behaviors when left alone at home.

## Simple Summary

This study addresses the problem of abnormal canine behavior detection when dogs are left alone at home, where existing visual methods struggle with complex temporal changes and a lack of labeled data. The aim is to develop an effective intelligent detection model using video data and deep learning. The work constructs a new dog behavior dataset and proposes a model with two key modules to better capture time-related features and distinguish normal from abnormal behaviors. The results show that the model achieves high detection accuracy and outperforms existing methods. The findings provide a reliable non-contact way to monitor dog behaviors, which helps improve pet welfare, reduce owners’ worries, and support smart home and animal care applications.

## 1. Introduction

Canines are among the most important companion animals for human beings and can provide emotional and social support. However, when left at home alone, dogs often experience anxiety, which can lead to various behavioral abnormalities, such as excessive barking, destructive behaviors, and indoor elimination. Identifying these abnormal behaviors and implementing scientifically sound interventions can help improve canine welfare and promote harmonious coexistence between humans and companion animals.

Traditional methods for monitoring dog behavior typically rely on wearable sensor devices to collect data and analyze dogs’ behaviors and physiological status [1,2,3]. However, these methods have significant limitations. First, wearable monitoring devices and collars with improper tightness or fixation methods may alter dogs’ natural movements and shift sensor positions. These issues will introduce measurement deviations and unreliable data during long-term monitoring [4,5]. Second, such devices primarily focus on physiological indicators (e.g., heart rate and activity levels) or location data, which tends to limit their capacity to identify various canine abnormal behaviors [2,3]. Third, wearable devices require regular charging and maintenance, entailing high costs, and thus fail to meet long-term, convenient monitoring needs [6].

Video Anomaly Detection (VAD), a hot research topic in the field of computer vision, enables the automatic identification and localization of anomalous events by analyzing abnormal patterns in video sequences. It has been widely applied in fields such as security surveillance and behavioral analysis [7,8]. With the increasing prevalence of surveillance cameras in homes, the adoption of video-based, non-contact behavioral detection has emerged as a potential solution for ensuring the welfare of domestic dogs. However, due to the scarcity of data on abnormal behaviors in domestic dogs and the lack of domain-specific pre-trained weights in the field of canine behavior, the effectiveness of transfer learning is limited, and models are prone to overfitting [9].

Abnormal behaviors in domestic dogs are characterized by their scattered and diverse nature; performing frame-level annotation of such behaviors requires significant investment of labor. Semi-supervised methods employ the way of “training a model to learn normal behavioral patterns and classifying behaviors that deviate from these patterns as abnormal” [7,10,11]. Although these methods avoid costly annotation, their application to abnormal behavior detection in domestic dogs still faces significant limitations. On the one hand, models are prone to misclassifying unseen normal behaviors as abnormal. This is primarily due to highly diversified normal behaviors and distinct behavioral patterns exhibited by individual dogs, both of which lead to the impracticality of collecting all possible normal behavior videos for model training. On the other hand, semi-supervised methods do not incorporate any prior knowledge regarding abnormal behaviors in domestic dogs and rely solely on normal behavior videos to train the model, making it inherent difficult to precisely define the boundary between “normal” and “abnormal” [7].

More recently, the focus has shifted toward weakly supervised learning methods [8,12,13,14,15]. Weakly Supervised Video Anomaly Detection (WSVAD), where only video-level binary labels (i.e., normal vs. abnormal) are available, each video sequence is partitioned into multiple snippets. Hence, all the snippets are normal in a normal video, and at least one snippet contains the anomaly in an abnormal one [8]. These methods have three key advantages: (1) They utilize both normal and abnormal videos during the training process, which allows for more nuanced and discriminative learning of what differentiates the two. (2) They simplify the annotation process by only requiring labels that confirm the presence of an anomaly, without needing to pinpoint its exact occurrence. (3) They make it feasible to compile larger datasets, as the annotation requirements are considerably less stringent [16].

Hence, this study aims to develop a weakly supervised model for recognizing abnormal behaviors in domestic dogs. The main innovations include the following three points:

(1) To address the lack of datasets in the field of abnormal behavior recognition in domestic dogs, we collected videos from the official standard DouYin and constructed a domestic dog behavior dataset (Alone-Dog).
(2) We introduce a Temporal Context Aggregation (TCA) module that enhances VAD by employing a reused attention matrix and adaptive context fusion. It captures both local and global dependencies simultaneously and enhances the model’s ability to represent temporal behavioral features of canines.
(3) To address the issue of blurred boundaries caused by “abnormal behaviors being embedded within a large amount of normal behavior,” the Pseudo-Instance Discriminative Enhancement (PIDE) module is introduced to enhance the model’s ability to distinguish abnormal behaviors.

## 2. Related Work

With regard to canine behavior recognition, current mainstream technologies can be categorized into three types: contactless, wearable, and multimodal fusion. Each type relies on different sensing mechanisms and fits different scenarios. A detailed overview of research on canine behavior based on these different technologies is provided in Table 1.

**Contactless recognition:** Contactless recognition relies on visual perception technology and does not require dogs to wear additional devices, making it the preferred solution for long-term monitoring and detailed analysis of domestic dog behavior in the home and other environments. Schork et al. [17] developed a BlyzerDS system based on convolutional neural networks (CNNs), which uses infrared surveillance video to automatically detect dogs’ sleep duration and sleep segmentation, achieving a consistency of up to 89% compared with manual observations. DigiDogs [18] utilized synthetic training data to estimate canine 3D poses, supporting low-cost, point-of-interest-free full-body key point detection. This way effectively alleviates the scarcity of real-world labeled data, with a PCK (Percentage of Correct Keypoints) of 83.8%. Martin et al. [19] extracted 3D tail positions using Mask R-CNN and derived temporal metrics such as velocity and momentum. In an experiment involving 30 domestic dogs, researchers validated the correlation between tail movements and emotional expressions under various stimuli.

In addition to quantitative studies of canine behavior, there have also been qualitative analyses of domestic dog behavior. Bleuer-Elsner et al. [20] used their proprietary K9-Blyzer visual tracking tool to extract movement features, identifying three movement pattern dimensions associated with canine Attention Deficit and Hyperactivity Disorder (ADHD)-like behavior (high-speed movement, extensive spatial coverage, and frequent turning). This thereby provides a basis for the objective diagnosis of neurobehavioral abnormalities. Stephan et al. [21] observed the behavior of domestic dogs left alone using home panoramic video and found that domestic dogs exhibited higher activity levels when in the presence of familiar conspecifics. Male domestic dogs were more likely to linger near doorways and vocalize more frequently, which establishes a real-world baseline for separation-related behaviors.

With the explosive growth in the volume and complexity of behavioral data in the emerging field of canine science, another study [22] simulated canine behavior and aimed to plan a dog’s movement. It used datasets of first-person video footage captured from the dog’s perspective and their corresponding actions as input to train their model. Farhat et al. [23] systematically reviewed the research framework, proposing a three-stage process involving behavioral quantification, feature extraction, and predictive analysis, to assess the existing landscape of automation applications in canine science for dog behavior analysis. They thought current studies should strengthen the understanding of innovative technologies, weaken interdisciplinary barriers, and reduce high learning costs to realize the practical implementation of these technologies. The above-mentioned studies lay the foundation for future long-term monitoring of domestic dogs.

**Wearable Recognition:** Compared with non-contact visual detection, wearable detection relies on miniature sensors to enable round-the-clock and high-precision collection of movement and physiological signals. This makes it particularly suitable for accurately capturing subtle behaviors and continuous changes in physiological states. Most studies [2,3] attached various monitoring devices to canines’ necks or backs, focusing solely on the classification of normal behaviors such as eating, drinking, and sitting. A small number of studies [24] incorporated behaviors that may indicate physiological abnormalities in domestic dogs (such as licking and rubbing) into their classification tasks, while other studies [25] focus exclusively on the identification of a specific type of abnormal behavior, for example, pruritic behaviors.

Junhyeok Go et al. [2] proposed a semi-supervised learning framework to recognize dogs’ behaviors using data collected by wearable sensors. In order to address the issue of high annotation workload, they introduced an embedding space distance loss on top of Meta Pseudo Labels (MPL) to automatically identify and filter out invalid data, while integrating unsupervised data augmentation (UDA) to improve the quality of pseudo labels. Experimental results demonstrated that the proposed method with a small amount of annotated data achieves an accuracy of 88.31%, which significantly outperforms supervised learning and conventional MPL methods. This provides an effective solution for temporal behavior recognition in domestic dog sensing under resource-constrained scenarios.

**Multimodal Detection:** As a cutting-edge research direction, multimodal fusion detection can infer from occlusion, lighting conditions, and motion noise by integrating data from multiple sources. Khush Attarde et al. [26] constructed a dataset consisting of 923 real-world canine video clips and 7120 question–answer pairs to perform frame-level multimodal reasoning and cross-modal cue fusion for fine-grained analysis tasks such as canine behavior, emotion, and social interaction. They were the first to integrate synchronized audio-video modalities in this field. Kim et al. [1] proposed a multimodal behavior recognition method that fused visual data from home cameras with data from neck-worn wearable sensors, enabling the identification of seven categories of daily behaviors with an accuracy of 93.4%. This method, fused with other types of data, can improve accuracy, and if data noise occurs, it can be compensated for. Tran [9] proposed an audio-video multimodal recognition framework for canine social signal classification to solve the problems of few samples and high noise. Additionally, they explored cross-domain transfer learning and self-supervised pre-training to enhance the model’s acoustic feature representation capability under low-data-sample scenarios.

**Table 1:** Studies on canine behavior using different techniques.

Techniques	Year	Objective	Data Acquisition Equipment	Dataset Specification	Model Used	Result (Accuracy)	Limitations
Contactless Detection	2018 [22]	It models canine behaviors and movements (including future motion prediction and action sequence planning), and achieves generalizable visual feature transfer.	a GoPro camera on the dog’s head	Training set: 21,000 frames Validation set: 1500 frames Test set: 2000 frames	ResNet-18 LSTM	Acting: 21.62% Planning: 19.77%	Only visual data were considered.
	2022 [1]	It recognizes seven types of canine behaviors: standing, sitting, lying on back with head up, lying on back with head down, walking, sniffing, and running.	IP camera, three-axis accelerometer and three-axis gyroscope	Training set: 668 Videos Test set: 167 Videos	YOLOv4 CNN-LSTM	93.4%	The sensor is mounted at a fixed position (around the neck) and is sensitive to head motions.
	2024 [17]	It enables contactless monitoring of canine sleep behavior, including sleep duration and the number of sleep episodes.	Swann SWDVK-845504	Training set: 13,668 Videos (80,000 frames) Test set: 10 Videos (6000 frames)	BlyzerDS	89%	The BlyzerDS system demands extensive video monitoring to ensure efficient and accurate results.
	2024 [18]	It estimates 3D canine pose from single-view RGB images, alleviating the scarcity of real-world 3D datasets and mitigating depth ambiguity.	Grand Theft Auto (GTA)	118 Videos (27,900 frames)	D-Pose	PCK@0.15 = 83.8%	The quantitative results indicate the existence of a domain gap in the Z-coordinate space.
	2024 [19]	It performs 3D pose tracking and quantitative analysis of canine tail wagging.	RealSense D415 depth camera	300 + 2500 Images	Mask R-CNN	Tail: 67.9% Base of tail: 64.1% End of tail: 38.5%	The system is susceptible to errors arising from simplifying assumptions or random factors.
Wearable Detection	2018 [25]	It identifies itching behaviors in dogs, including scratching and head-shaking.	AX3 data logger, Cannon VIXIA HF R600 GoPro Hero4	112,082 Labeled frames	EMADE	Shaking: 72.16% Scratching: 76.85%	Behaviors such as paw licking and body rubbing for alleviating itching are not addressed in this work.
	2021 [24]	It recognizes seven types of canine behaviors: eating, drinking, licking, petting, rubbing, scratching, and sniffing.	Whistle FIT^®^ Whistle 3^®^ Whistle GO^®^	163,110 samples	FilterNet	Eating: 98.8% Drinking: 94.9% Licking: 77.2% Petting: 30.5% Rubbing: 72.9% Scratching: 87.0% Sniffing: 61.0%	This study relied solely **on motion signals**.
	2024 [2]	It accurately recognizes five types of canine behaviors: standing, walking, sitting, lying down, and eating.	IMU (ICM-20948) MCU (MAX32 670GTL) PMU (MAX77734)	Labeled Data: 9487 samples Unlabeled Data: 11,008 samples	ResNet	88.31%	Sensor data may be affected by the comfort level of the canine wearing the device.
	2025 [3]	It recognizes seven types of canine behaviors: dashing, sitting, standing, trotting, walking, lying on the chest, and sniffing.	Kaggle Dataset ActiGraph GT9X Link	6,035,746 samples	Hybrid Cascading Model (HCM)	96.82%	HCM is highly complex, requires lengthy training times, and consumes substantial computational resources.
Multimodal Detection	2025 [9]	It recognizes multimodal audiovisual social signals in canines under few-shot conditions.	Audio Set YouTube	550 Labeled Videos 9994 Audios	AST S3D JFM	55.3%	Limited training data and high noise levels, severe class imbalance.
	2025 [26]	It establishes a fine-grained multimodal benchmark for understanding canine behavior.	YouTube	7120 Q&A pairs from 923 videos	MLLMs	Gemini2.0-Flash: 61.07% GPT-4o: 56.07%	Reliance on closed-source models, high annotation costs, insufficient samples of rare behaviors

## 3. Materials and Methods

### 3.1. Materials

#### 3.1.1. Data Collection

Data used in this study were collected from DouYin, a popular short-video platform. Behavior-related keywords, including “dog left home alone surveillance video”, “dog quietly at home surveillance”, “dog destroying furniture surveillance”, “puppy left home alone surveillance” and “puppy wreaking havoc at home surveillance”, etc., were used to search for dog behavior videos. After downloading, data were excluded if they met any of the following criteria: videos captured by handheld cameras, grainy videos, severely shaky camera shots, speed-accelerated videos, and videos depicting human-dog interaction.

Ultimately, a total of 642 high-quality videos were collected, comprising 366 videos of abnormal behaviors and 276 videos of normal behaviors. The video duration distribution in our dataset shows a pronounced concentration pattern: videos under one minute account for 89%, those between one and two minutes make up 10%, while only 7 videos (less than 1%) exceed two minutes in length.

#### 3.1.2. Definition of Abnormal Behavior in Dogs

**Figure 1:**
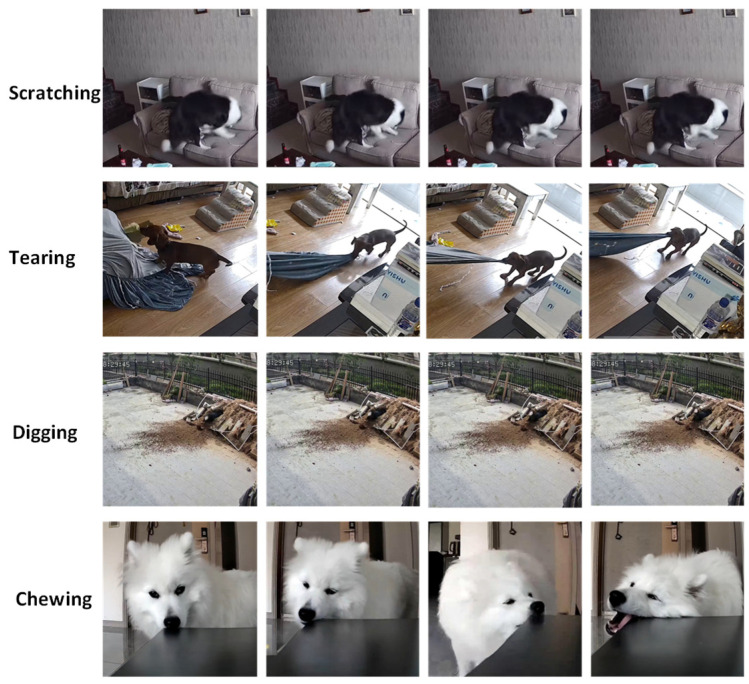
Examples of different anomalies in our dataset. (All images in the figures are screenshots captured from videos collected from Douyin, and all relevant permissions have been obtained from the pet owners).

Generally speaking, abnormal behavior refers to dysfunctional and unusual behavioral patterns [27]. Dogs often exhibit anxiety after being left alone for about 15 to 30 min, and manifest behaviors such as kennel destruction, wall or door damage, excessive barking, indoor defecation [27]. Although such behaviors are psychologically or physiologically normal responses, this study focuses on undesirable behaviors exhibited by dogs when left unattended. Therefore, the aforementioned behaviors are classified as abnormal. Based on this definition, the abnormal behaviors of dogs targeted in this study are presented in Table 2, mainly including four types: scratching, tearing, digging, and chewing. Four-frame images of these four abnormal behaviors are shown in Figure 1.

#### 3.1.3. Data Annotation

To avoid labor-intensive labeling workload for data, we adopt a weakly supervised approach based on the Multiple-Instance Learning (MIL) [28,29,30], which uses weakly labeled training videos. Specifically, training labels (abnormal or normal) are assigned at the video-level rather than the clip-level. During training, both normal and abnormal behavior videos are used simultaneously, with each video treated as a “bag” and its segments as “instances” within the bag. Hence, we only need to perform video-level annotation on the training set: videos containing abnormal behavior instances are labeled as “abnormal”, and those without such instances are labeled as “normal”.

**Figure 2:**
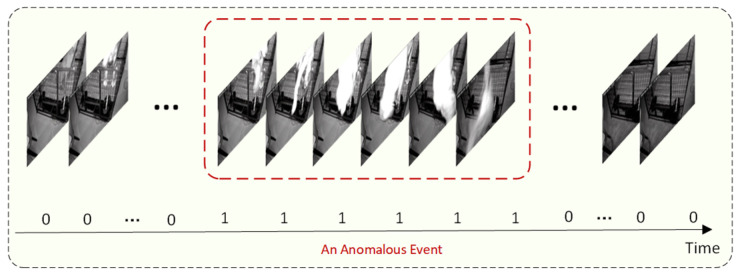
An example of labeling results for the testing set.

To evaluate the model’s performance on test videos, frame-level annotations are required for abnormal events in each video, specifically the start and end frames of each abnormal behavior segment. The annotation was conducted by examining frame sequences using LabelImg 5.4.1 and Python scripts. Frames involving abnormal behavior were labeled as “1”, whereas frames with normal behavior were labeled as “0”. A detailed annotation example is presented in Figure 2, which shows a complete anomalous event, referred to as “instance”. Finally, a Python script running on Python 3.8.10 and Jupyter Notebook 6.4.4 was employed to convert the annotated results into a structured format file.

Finally, the dataset contains a training set and a testing set. The training set consists of 200 normal behavior videos and 230 abnormal behavior videos, while the testing set comprises the remaining 30 normal videos and 30 abnormal videos. In the testing set, normal videos contain only normal frames, with a total of 22,240 frames. While for abnormal videos, there are 10,943 normal frames and 12,337 abnormal frames. Detailed statistics are provided in Table 3.

### 3.2. Methods

#### 3.2.1. Overall Framework

**Figure 3:**
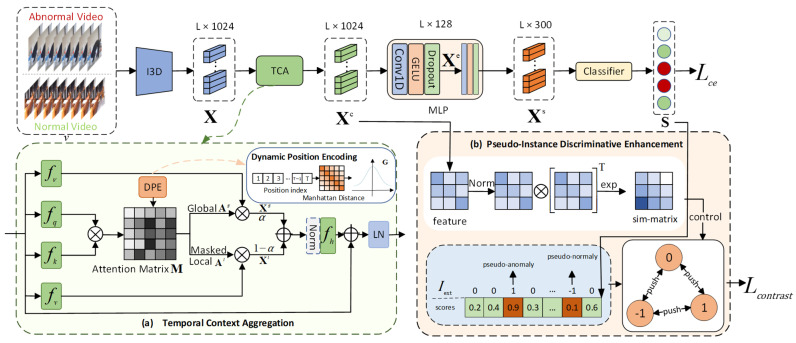
Overall framework of the TP-CanineNet.

The overall framework of the proposed TP-CanineNet model is illustrated in Figure 3. Specifically, untrimmed videos are initially divided into non-overlapping clips by a sliding window of 16 frames, and then each clip is fed into an Inflated 3D Convolutional Network (I3D) [31] to extract optical flow features, represented by X. Optical flow features X were sent into the TCA module, as displayed in Figure 3a, to generate context features $X^{c}$. After that, feature reduction is achieved through a two-layer multi-layer perceptron (MLP). Finally, the classifier predicts clip-level anomaly score **S**. During the training phase, the MIL-based loss function $L_{ce}$ is utilized to aggregate clip-level prediction scores into video-level outputs, enabling the model to concentrate on key frames with high responses associated with anomalous behaviors. PIDE module, as shown in Figure 3b, took $X^{c}$ as input and applied to the middle layer to learn highly discriminative features of abnormal and normal behaviors from $X^{c}$ under the guidance of pseudo-labels $I_{ext}$.

#### 3.2.2. Optical Flow Feature Extraction for Canine Behavior

In this study, a pre-trained I3D backbone network on the Kinetics-400 video dataset [31] is adopted in the training phase to extract the optical flow feature. Given an input clip *v* with sequence length L, 5-crop is applied to generate patches from the top-left, top-right, bottom-left, bottom-right, and center regions of each clip. The augmented video clips are fed into the I3D model to extract optical flow features X $\in R^{L\times D}$, where D = 1024 represents the feature dimension.

#### 3.2.3. Temporal Context Aggregation Module

To address the limitations of insufficient long-range dependency modeling and the lack of local context information in temporal feature representation, this study introduces a TCA module that is different from conventional multi-branch parallel structures adopted in existing approaches. TCA module, illustrated in Figure 3a, explicitly models global–local dependencies via joint attention matrices and adaptive feature fusion.

First, the optical flow feature X of the clip is projected to the latent space by different linear layers. The attention matrix **M** is subsequently obtained by inner product calculation, as formulated in Equations (1) and (2):

(1) $$f_{q}=XW_{q},\;f_{k}=XW_{k},\;f_{v}=XW_{v}$$

(2) $$M=f_{q}\cdot {f_{k}}^{⊤}$$

where $W_{q},W_{k},W_{v}\in R^{D\times D}$ are learnable projection matrices to linearly project the input feature into query, key and value spaces in our attention module, $f_{q},f_{k}$ and *f*_v_ denote three independent linear layers, ⊤ refers to the transpose operation, and D = 1024 represents the feature dimension.

In addition, considering the importance of position information, we introduce Dynamic Position Encoding (DPE) to model the relative distances of clips, formulated as:

(3) $$G_{\mathrm{ij}}=\exp(-|\gamma (i-j{)}^{2}+\beta |)$$

where i and j denote the absolute positions of two clips, and γ and β are learnable weights and bias terms. γ controls the decay rate of positional correlation with the temporal interval, while β serves as a learnable offset to adjust the lower bound of position weights. In particular, the DPE is embedded into the attention matrix **M** as a location prior, i.e., **M** ← **M** + **G**, thus avoiding affecting the original feature distribution. Unlike fixed position encoding in [32], DPE adapts to varying video lengths due to its dynamic nature. Also, the Gaussian-like kernel of DPE inherently suppresses the influence of long-distance noise, which emphasizes closer clip relationships over distant ones, implying an innate sensitivity to non-linear patterns.

Softmax normalization is then applied to generate the global attention map $A^{g}$. The projected clip features are re-weighted by the attention map to obtain the global context features $X^{g}$, as formulated below:

(4) $$A^{g}=softmax\left(\frac{M}{\sqrt{D_{h}}}\right)$$

(5) $$X^{g}=A^{g}\cdot f_{v}$$

where $D_{h}$ is the hidden dimension in the latent space.

Although the above operation facilitates global context modeling, it inevitably introduces long-range noise. To address this issue, we implement local context calibration by reusing the attention matrix with a masking window, which can be formulated as:

(6) $${\tilde{M}}_{ij}=\left\{\begin{array}{ll} M_{ij}, & if\;j\in [\max(0,i-\lfloor \frac{w}{2}\rfloor ),\min(i+\lfloor \frac{w}{2}\rfloor ,L)]\\ -\infty , & otherwise \end{array}\right.$$

where *w* is the window size of the mask and L is the maximum length of the input sequence. Equation (6) ensures that the i-th clip interacts only with its neighborhood of window size *w*. The lower bound of this window is the earliest moment that can be historically observed, and the upper bound is the maximum length of the sequence. Similarly, the projected clip feature is re-weighted with the attention map $A^{l}$ to obtain local calibration features $X^{l}$, which can effectively capture slight changes and achieve feature enhancement in the local neighborhood, as formulated in Equations (7) and (8):

(7) $$A^{l}=softmax\left(\frac{\tilde{M}}{\sqrt{D_{h}}}\right)$$

(8) $$X^{l}=A^{l}\cdot f_{v}$$

Subsequently, we resort to a learnable fashion rather than direct concatenation or average pooling to achieve local-global contextual adaptive fusion, allowing the model to dynamically balance the importance of global temporal patterns and local nuances. The fusion process is formulated as follows:

(9) $$X^{o}=\alpha \cdot X^{g}+\left(1-\alpha \right)\cdot X^{l}$$

(10) $$X^{c}=LN\left(X+f_{h}\left(Norm\left(X^{o}\right)\right)\right)$$

where α and 1 − α denote the global and local weight for context fusion, respectively, and Norm(·) denotes a combination of power normalization [32] and L2 normalization. Then a linear layer $f_{h}\left(\cdot \right)$ followed by residual connection and layer normalization LN(·) is applied to obtain context feature $X^{c}\in R^{L\times D}$.

#### 3.2.4. Multilayer Perceptron and Classifier

To obtain high-level semantic representations, a two-layer MLP is utilized for feature reduction. Each Conv1D layer is followed by a Gaussian Error Linear Unit (GELU) activation and dropout operation. This process is denoted as follows:

(11) $$X^{e}=Dropout\left(GELU\left(Conv1D\left(X^{c}\right)\right)\right)$$

(12) $$X^{s}=Dropout\left(GELU\left(Conv1D\left(X^{e}\right)\right)\right)$$

Finally, a causal convolution layer is employed to predict clip-level anomaly scores, which considers both current and historical observations to obtain more reliable results. The classifier is formulated as:

(13) $$S=\sigma \left(f_{t}\left(X^{s}\right)\right)$$

where $f_{t}\left(\cdot \right)$ is the causal convolution layer with kernel size ∆t = 3, σ(·) is the sigmoid function, and $s_{i}\in \left\{s_{1}\ldots s_{\left\lfloor L/16+1\right\rfloor }\right\}$ is the anomaly score of the *i*-th clip.

#### 3.2.5. Pseudo-Instance Discriminative Enhancement Module

The PIDE module, whose structure is illustrated in Figure 3b, is designed to alleviate the misclassification between normal behaviors and ambiguous abnormal behaviors of canines. Based on a feature optimization scheme via extreme-instance contrastive learning, this module performs contrastive learning on the extreme instances with high anomaly scores, which can be acquired from **S**. Through this, PIDE enables to accurately capture of the representative core features of abnormal events and effectively discriminates anomalies related to canine abnormal behaviors from complex normal contexts. The feature representation of the optimized extreme instances is formulated in Equation (14):

(14) $$Idx_{pa}^{\left(b\right)}=\{\underset{i\in \left\{1\ldots T_{b}\right\}}{\mathrm{argmax}}\{s_{i,b}\}\},\;Idx_{pn}^{\left(b\right)}=\{\underset{i\in \{1\ldots T_{b}\}}{\mathrm{argmin}}\{s_{i,b}\}\}$$

Specifically, $Idx_{pa}^{\left(b\right)}$ denotes the temporal index of the instance with the maximum anomaly score $s_{i,b}$ within the b-th video sample in a training batch, which is selected as the positive anchor sample for contrastive learning. Correspondingly, $Idx_{pn}^{\left(b\right)}$ refers to the index of the instance with the minimum anomaly score, treated as the negative sample. On the basis of clip-level anomaly scores **S** predicted by Equation (13), we further construct the set of selected extreme instances within each batch as follows:

(15) $$I_{ext}=\cup_{b}\left\{\left(b,i\right)|i\in Idx_{pa}^{\left(b\right)}\vee i\in Idx_{pn}^{\left(b\right)}\right\}$$

We take the union over all video samples b and collect all $\left(b,i\right)$ pairs where the time segment index i is either the positive anchor (from $Idx_{pa}^{\left(b\right)}$) or the negative counterpart (from $Idx_{pn}^{\left(b\right)}$) of the *b*-th video.

Furthermore, to regularize the feature distribution and enhance the stability of subsequent contrastive learning, the features $X^{c}$ output by the TCA module are directly normalized via L2 normalization as follows:

(16) $${\tilde{X}}_{b,\;i}^{c}=Norm\left(X_{b,\;i}^{c}\right),\;\forall \left(b,\;i\right)\in \;I_{ext}$$

Such normalization alleviates the interference of feature amplitude variations on discriminative feature learning, thus facilitating more robust loss computation in subsequent steps.

#### 3.2.6. Loss Function

We adopt the MIL-based loss as the basic objective function. Specifically, we determine the video-level prediction $\tilde{y}$ by taking the mean value of the top-*k* anomaly scores. For positive bags, we set $k=\left\lfloor \frac{L}{16}+1\right\rfloor$, and for negative bags, we set *k* = 1. Given a batch containing B samples with video-level ground-truth $y_{i}$, the binary cross-entropy is formulated as:

(17) $$L_{ce}=\sum_{i=1}^{B}-y_{i}\log({\tilde{y}}_{i})$$

In the PIDE module, the supervised contrastive loss is adopted to impose contrastive learning constraints across instances. For the normalized features ${\tilde{X}}^{c}=\left\{{\tilde{X}}_{b,\;i}^{c}|(b,\;i)\in I_{ext}\right\}$, the contrastive loss term is formulated as follows when $\left|P\left(i\right)\right|>0$:

(18) $$L_{contrast}^{\left(i\right)}=-\sum_{p\in P(i)}\frac{1}{\left|P(i)\right|}\log\frac{\exp({{{\tilde{X}}^{c}}_{i}}^{T}{{\tilde{X}}^{c}}_{p}/\tau )}{\sum_{k\in A(i)}\exp({{{\tilde{X}}^{c}}_{i}}^{T}{{\tilde{X}}^{c}}_{k}/\tau )}$$

We define $A\left(i\right)=I_{ext}/\left\{i\right\}$ and $P\left(i\right)=\left\{p\in A\left(i\right)|y_{p}^{pseudo}=y_{i}^{pseudo}\right\}$, where $y_{b,\;i}^{pseudo}\in \left\{+1,-1\right\}$ denotes the pseudo-label, ${{\tilde{X}}^{c}}_{i}$ represents the anchor feature, and τ is the temperature coefficient.

Subsequently, a push mechanism is introduced to actively propagate information from the anchor features to their local neighbors. By aggregating neighboring features via similarity-weighted averaging, we suppress feature magnitude variations and enhance local feature correlations, thus improving the model’s capacity to capture local key features.

The final batch-level loss is obtained by averaging all valid anchor losses $L_{contrast}^{\left(i\right)}$ within the batch:

(19) $$L_{contrast}=\frac{\sum_{i\in I_{ext}}I(|P(i)|>0)\cdot L_{contrast}^{\left(i\right)}}{\sum_{i\in I_{ext}}I(|P(i)|>0)+\epsilon }$$

where $I\left(\cdot \right)$ denotes the indicator function, which equals 1 if the corresponding condition holds and 0 otherwise. ϵ = $10^{-8}$ represents a small constant to avoid division by zero.

During the training stage, the overall objective function of the model is formulated as:

(20) $$L=L_{ce}+\lambda L_{contrast}$$

where λ is a coefficient that balances the contributions of the classification loss and the contrastive loss. By optimizing this objective function, the proposed model is able to learn discriminative representations for positive and negative samples, thereby effectively alleviating misclassification between normal and abnormal behaviors of dogs.

## 4. Experimental Results

### 4.1. Experiment Environment

All experiments in this study were implemented on the AutoDL cloud computing platform, equipped with an AMD EPYC 9754 128-core CPU, an NVIDIA RTX 4090D GPU with 24 GB memory, and Ubuntu 20.04 LTS. The implementation was developed in Python 3.8 with the PyTorch 1.8.1 deep learning framework and CUDA 11.1.

During training, we used Adam as optimizer, and the batch size was 128 and the maximum epochs were 50. The initial learning rate was set to 5 × 10^−4^, and a cosine annealing learning rate decay strategy was applied to adjust the learning rate. To balance efficiency and performance, the maximum sequence length of training clips was set to 400. Random seed was fixed at 4 to ensure reproducibility.

### 4.2. Evaluation Metric

We adopted frame-level area under the precision-recall curve (AUPRC, or average precision (AP)), false alarm rate (FAR), and area under the receiver operating characteristic curve (AUC) as evaluation metrics [15,33].

AP is computed as the area under the precision-recall curve, as formulated in Equation (21):

(21) $$AP=\int_{0}^{1}P\left(r\right)dr$$

where P(r) denotes the precision under a given recall level *r*. Due to the clear discriminability between normal and abnormal frames, AP serves as a key metric for anomaly detection. In contrast to AUC, which characterizes overall classification performance, AP focuses more on the quality of anomaly detection and yields a more reasonable and convincing evaluation of the model’s actual detection capability.

Furthermore, to ensure the reliability of anomaly detection, a false alarm rate (FAR) with a threshold value of 0.5 is employed for evaluation [34].

(22) $$FAR=\frac{FP}{FP+TN}$$

where FP denotes normal frames misclassified as anomalous, and TN denotes normal frames correctly identified. In anomaly detection, a lower FAR on normal videos indicates a more robust anomaly detection method [35].

### 4.3. Comparative Study

To validate the efficacy of TP-CanineNet, we compare our method with two classic weakly supervised video anomaly detection (WS-VAD) baselines, namely IFS-VAD [33] and MIST [34]. Both methods share a core design consistent with our TP-CanineNet: they rely on pseudo-label generation to mitigate the lack of fine-grained annotations under weak supervision.

Specifically, MIST constructs a multiple-instance pseudo-label generator with a sparse continuous sampling strategy to generate trustworthy clip-level pseudo-labels. IFS-VAD introduced a feature-similarity-based anomaly criterion, namely inter-clip feature similarity (IFS), to generate pseudo-labels with more reliability.

Notably, IFS-VAD makes use of the local–global temporal dependencies, which utilize Multi-scale Temporal MLP and different temporal strides through two branches. These key characteristics closely align with the core design principles of TP-CanineNet, making IFS-VAD and MIST comparable and suitable baselines for our evaluation.

The IFS-VAD and MIST models are trained on the Alone-Dog dataset under identical experimental conditions. AP is employed as the crucial comparison metric. The comparative results are presented in Table 4. The MIST method achieves an AP value of 62.94%, which is the lowest among all compared approaches. The IFS-VAD obtains a higher AP of 64.85%, outperforming MIST by 2.91 percentage points. Remarkably, our TP-CanineNet achieves the best AP performance (72.55%), surpassing MIST and IFS-VAD by 9.61% and 7.70%, respectively. The consistent feature extraction setting eliminates the interference introduced by different backbone networks, guaranteeing the fairness of the comparative experiment.

### 4.4. Ablation Studies

#### 4.4.1. Ablation Studies for Each Component

Table 5 summarizes the effects of each component in the proposed TP-CanineNet model. The incorporation of either the TCA module or the PIDE module independently sustains a stable AUC and significantly enhances the AP by 4.73% or 1.61% compared to the baseline model. The integration of both the TCA and PIDE modules leads to the optimal overall performance, demonstrating notable improvements in both the AUC, by 1.84%, and the AP, by 5.58%. Their collaborative integration effectively strengthens the feature representation capability and detection performance of the proposed model.

**Figure 4:**
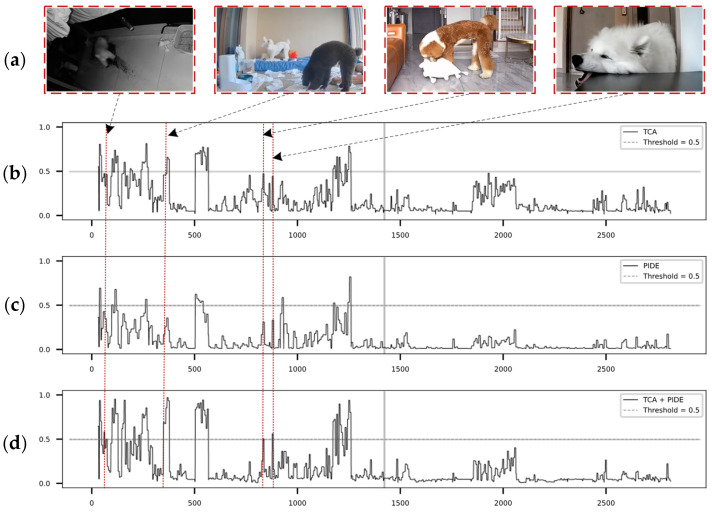
Visualization of anomaly scores of diverse module configurations. The horizontal axis represents the video frame index, and the vertical axis denotes the model’s predicted anomaly scores for the target behavior. (**a**) Four sample frames depict typical canine solitary behavior scenarios, and these scenes correspond to the moments indicated by the dashed lines. Subplots (**b**), (**c**), and (**d**) correspond to the model with only the TCA module, only the PIDE module, and the proposed full model with TCA and PIDE modules, respectively. (All images in the figures are screenshots captured from videos collected from Douyin, and all relevant permissions have been obtained from the pet owners).

To more effectively demonstrate the performance of our model, the anomaly scores obtained from the experiment are visualized in Figure 4. Figure 4a shows the behavior of the dog at four different points for comparison. Figure 4b,c show the anomaly scores after adding TCA and PIDE alone, respectively. Figure 4d shows the anomaly scores resulting from the integration of the above two modules. A comparison of these results shows that the TCA and PIDE together notably enhance the separability between normal and abnormal behaviors, leading to higher anomaly scores for abnormal clips and lower anomaly scores for normal clips. The four red dot lines show typical abnormal scores comparison of four time points. This indicates that after the integration of the TCA and PIDE modules, abnormal behaviors of dogs were more likely to be detected.

#### 4.4.2. Ablation Studies for Different Feature Backbones

Ablation studies were conducted to assess the impact of different pre-trained feature backbones on our training stage. Specifically, we utilized three feature backbones: I3D [31], S3D [32], and R (2 + 1) D [36]. In this study, validation experiments were separately conducted on the RGB feature and the optical flow feature extracted by the I3D feature backbone network. The results of these ablation studies are displayed in Table 6. Notably, the optical flow feature used by TP-CanineNet exhibited the optimal experimental performance, with an AUC of 85.19%, an AP of 72.55%, and a FAR of 0.00%. It gains 4.79% AUC and 7.94% AP over the strongest feature backbone, I3D-RGB, and reduces FAR from 1.73% to 0.00%.

**Figure 5:**
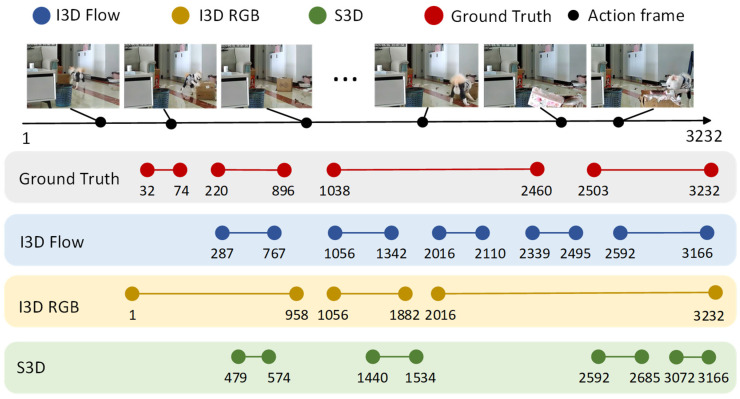
The performance of the TP-CanineNet with different feature backbones for the same video sample. (All images in the figures are screenshots captured from videos collected from Douyin, and all relevant permissions have been obtained from the pet owners).

In order to illustrate the effectiveness of optical flow extracted by I3D, we employed a video sample with 3232 frames, and all temporal intervals in which abnormal behavior occurs are marked. In this study, we used I3D and S3D to extract features. I3D can extract features from both optical flow and RGB of the video, while S3D extracts spatiotemporal features of the video. Figure 5 depicts the experimental outcomes. It indicates that the features extracted by S3D are incapable of representing anomalies at numerous temporal locations, as the recognition results are not good enough, for instance, from frames 1534 to 2560. TP-CanineNet, using RGB features extracted by I3D, misclassifies a substantial number of normal clips as abnormal, such as from 2460 to 2503. Conversely, the Flow features yield significantly superior detection results.

### 4.5. Parameter Evaluation

In the TCA module, we employed an adaptive weight to fuse global and local features. We set the weights in two ways: fixed and learnable. Table 7 shows the results of fusion weights using fixed and learnable methods. It can be seen that the optimal performance is obtained with a global–local weight ratio of 1:1 in a fixed way. Therefore, the initial ratio for adaptive fusion is also set to 1:1, which facilitates the dynamic fusion of local and global contexts and enables better adaptation to diverse video content.

In the TCA module, *w*, a masking window size is employed to mitigate long-range noise. Therefore, we examine the impact of the window size on model performance in Table 8. Experimental findings suggest that the model attains optimal performance when *w* is 9, with an AUC of 85.19% and AP of 72.55%, and FAR is reduced to 0.00%. Smaller windows are unable to capture an adequate spatiotemporal context, whereas larger windows introduce an excessive amount of long-range noise, thus leading to low and unstable detection accuracy. Table 9 compares the detection performance of the Flow feature under different thresholds. It can be observed that the Flow feature achieves the best overall performance at tIoU = 0.5, with a FAR of 0 and no performance degradation.

**Figure 6:**
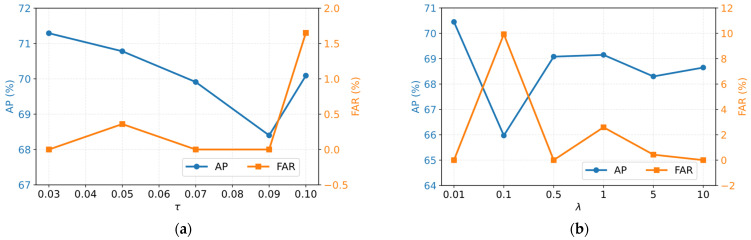
Model performance under different hyper-parameter settings. (**a**) and (**b**) show the AP and FAR results of our model under different temperature coefficients *τ* and loss weights *λ*, respectively.

In addition, we conduct an extensive exploration of the hyper-parameter configuration for temperature coefficients *τ* and loss weights *λ*, and the results are shown in Figure 6. For *τ*, the corresponding results are displayed in Figure 6a. As observed, AP reaches its maximum value of 71.29%, and FAR, for which a lower value indicates a more desirable effect, reaches its minimum value of 0.0% when *τ* = 0.03. In Figure 6b, AP and FAR exhibit a clear inverse relationship for *λ*. At *λ* = 0.01, AP achieves a global maximum of 70.45% with a FAR of 0.0%, demonstrating that this setting ensures both high detection accuracy and an extremely low FAR. When *λ* ≥ 0.5, AP remains relatively stable in the range of 68–69%, and FAR also stays at a low level, indicating that the overall performance tends to converge.

## 5. Discussion

### 5.1. Limitations of Existing Canine Behavior Recognition Methods

Nearly all existing canine behavior recognition systems are framed under a normal behavior classification paradigm, rather than the video anomaly detection task targeted in this domain. Representative works [1,2,3,24] only perform fine-grained categorization of predefined daily normal behaviors. A minority [17,25] only target a narrow abnormal category such as sleep and itching behaviors. These models uniformly adopt multi-class classification architectures that require exhaustive labeling of all normal behavioral categories, and they lack dedicated designs to distinguish abnormal, destructive behaviors (e.g., scratching, digging, chewing) from routine dog activities. Prior methods cannot be directly deployed for unsupervised or weakly supervised abnormal behavior alerting.

In stark contrast, our TP-CanineNet is specially constructed for canine abnormal behavior detection under weak supervision, filling the research gap left by existing classification-oriented systems. The embedded TCA module captures temporal behavioral features of canines and suppresses long-range noise, while the PIDE module introduces pseudo-instance contrastive learning to explicitly enlarge the feature discrepancy between normal and abnormal samples. Joint optimization of the two modules enables our model to reach a frame-level AUC of 85.19% and AP of 72.55% on the self-built Alone Dog dataset, with FAR reduced to 0.00%.

### 5.2. Overall Performance and Module Effectiveness

Experimental results demonstrate that the proposed TP-CanineNet achieves better performance for abnormal behavior detection on the self-constructed Alone-Dog dataset. Relative to the baseline model, it improves AUC by 1.84% and AP by 5.58%, and reduces FAR to 0.00%, verifying its effectiveness under weak supervision. While IFS-VAD is capable of multi-scale temporal modeling, it fails to yield sufficient temporal feature representation and exhibits weak normal–abnormal discriminability, which restricts detection performance.

TP-CanineNet’s performance gains arise from synergistic optimization of the Temporal Context Aggregation (TCA) and Pseudo-Instance Discriminative Enhancement (PIDE) modules. By reusing attention matrices, incorporating dynamic position encoding, and enabling adaptive local–global fusion, TCA captures long-range temporal dependencies and suppresses noise, significantly enhancing canine temporal feature representation; standalone TCA improves AP by 4.73%. The PIDE module leverages extreme instance contrastive learning with pseudo-label supervision to strengthen normal–abnormal feature discriminability, mitigating boundary ambiguity and misclassification; standalone PIDE increases AP by 1.61%. Their integration yields a greater-than-additive performance gain, validating the architecture’s rationality.

Feature backbone comparisons confirm I3D optical flow features as optimal for canine behavior detection. Compared with R(2 + 1)D, S3D, and I3D-RGB, optical flow features improve AUC by 4.79% and AP by 7.94%, with a substantial reduction in FAR. This superiority stems from optical flow’s focus on motion trajectories and dynamics, which precisely match scratching, tearing, digging, and chewing, while mitigating RGB’s susceptibility to illumination and pose variations. Parameter sensitivity analyses validate learnable global–local fusion weights and a 9 × 9 masking window as optimal for TCA, aligning with canine abnormal behavior temporal spans. Contrastive learning hyperparameters τ = 0.03 and λ = 0.01 balance feature discriminability and primary-auxiliary task weights, ensuring stable, reproducible results.

### 5.3. Limitations and Future Work

Several limitations are acknowledged. The dataset, sourced from short-video platforms, is restricted to indoor home environments with limited diversity in dog breeds, ages, and camera viewpoints, requiring further validation of cross-scenario and cross-breed generalization. The dataset targets four abnormal behavior categories, excluding common anxiety-related behaviors (e.g., excessive barking, pacing), necessitating expanded task coverage.

Additionally, TP-CanineNet has 1.2073 M parameters. In the test with 45,520 frames, the total inference time is 4 s, with an average single-frame inference latency of 0.0879 ms. The model lacks lightweight optimization, and its real-time deployment capability has not been validated. Despite these constraints, the Alone-Dog dataset is pioneering, given the field’s annotation scarcity and absence of public benchmarks, and TP-CanineNet offers an effective non-contact intelligent detection solution for canine abnormal behaviors. Future work will expand dataset diversity, integrate multimodal cues (audio, prompt) to enrich feature representation, optimize architecture for edge deployment, and develop fine-grained abnormal behavior recognition to advance practical pet welfare monitoring applications.

## 6. Conclusions

In this study, we focused on abnormal behavior detection for dogs, aiming to accurately locate the temporal intervals of abnormal events in surveillance videos using weak supervision. We proposed TP-CanineNet, which integrated the TCA and PIDE modules. TCA can capture both local and global dependencies by sharing attention matrices, which not only effectively reduces the number of parameters and computational cost but also significantly boosts detection performance. PIDE, which employs positive-negative sample pairs with pseudo-labels to enhance feature discriminability, has robust performance in identifying abnormal and normal behaviors. Experimental results demonstrated that the proposed method achieved a frame-level AUC of 85.19% and AP of 72.55%, outperforming the baseline by 2.20% and 8.33%, respectively. Future work will focus on the fine-grained recognition of abnormal behaviors to better understand the patterns of different abnormal behaviors. Furthermore, we plan to incorporate multi-temporal data captured throughout the day to obtain a more comprehensive understanding of the activity and behavioral patterns of dogs.

## Figures and Tables

**Table 2:** Categories of abnormal behaviors.

Abnormal Behaviors	Description
Scratching	It scratches the upholstery and walls, resulting in permanent marks.
Tearing	It tears up tissues, sofas, clothing, and carpets, rendering them unusable.
Digging	It digs up the potting soil, disrupting the neatness of the environment.
Chewing	It chews on furniture legs, slippers, electrical cords, and cages, causing structural damage to these items.

**Table 3:** The dataset details.

Dataset	Behavior	Number of Videos	Video Duration	Number of Normal Frames	Number of Abnormal Frames
Training set	Normal	230	1.74 h	-	-
	Abnormal	200	1.64 h	-	-
Test set	Normal	30	0.25 h	22,240	0
	Abnormal	30	0.26 h	10,943	12,337

**Table 4:** Comparison between TP-CanineNet and IFS-VAD.

Method	Feature	AP (%)
MIST	I3D Flow	62.94
IFS-VAD	I3D Flow	64.85
**TP-CanineNet (Ours)**	I3D Flow	**72.5** **5**

**Table 5:** The results of ablation studies for each component (TCA, PIDE).

Baseline	TCA	PIDE	AUC (%)	AP (%)	FAR (%)
✓			83.35	66.97	0.00
✓	✓		83.44 (+0.09)	71.70 (+4.73)	0.00
✓		✓	83.38 (+0.03)	68.58 (+1.61)	0.00
✓	✓	✓	85.19 (**+1.84**)	72.55 (**+5.58**)	0.00

Note: “✓” indicates that the corresponding module is included in the model configuration.

**Table 6:** The results of ablation studies for different feature backbones.

Feature Backbones	AUC (%)	AP (%)	FAR (%)
R (2 + 1) D	72.42	47.37	5.40
S3D	78.35	57.33	2.30
I3D-RGB	80.40	64.61	1.73
**TP-CanineNet (Ours)**	**85.19 (+4.79)**	**72.55 (+7.94)**	**0.00**

**Table 7:** The global–local adaptive fusion weights in the TCA module.

Methods	Global	Local	AUC (%)	AP (%)
Fixed	0.1	0.9	82.66	70.22
	0.3	0.7	82.79	70.83
	**0.5**	**0.5**	**83.08**	**70.55**
	0.7	0.3	82.96	71.36
	0.9	0.1	80.83	70.04
Learnable	α	1 − α	**85.19**	**72.55**

**Table 8:** The results of TP-CanineNet for different masking window sizes *w*.

*w*	AUC (%)	AP (%)	FAR (%)
5	80.10	65.71	18.42
7	81.75	68.42	6.76
**9**	**85.19**	**72.55**	**0.00**
11	80.63	68.52	0.00
13	79.44	67.51	0.00
15	79.91	67.39	0.00

**Table 9:** Performance of the proposed method under Different Thresholds.

**tIoU**	0.1	0.2	0.3	0.4	**0.5**
**FAR (%)**	18.27	7.70	0.86	0.43	**0.00**

## Data Availability

Data available on request due to copyright restrictions from all video owners; the corresponding data cannot be made publicly available.
